# YOLO-FR: A YOLOv5 Infrared Small Target Detection Algorithm Based on Feature Reassembly Sampling Method

**DOI:** 10.3390/s23052710

**Published:** 2023-03-01

**Authors:** Xingang Mou, Shuai Lei, Xiao Zhou

**Affiliations:** School of Mechanical and Electronic Engineering, Wuhan University of Technology, Wuhan 430070, China

**Keywords:** infrared dim-small target detection, feature reassembly, YOLO

## Abstract

The loss of infrared dim-small target features in the network sampling process is a major factor affecting its detection accuracy. In order to reduce this loss, this paper proposes YOLO-FR, a YOLOv5 infrared dim-small target detection model, based on feature reassembly sampling, which refers to scaling the feature map size without increasing or decreasing the current amount of feature information. In this algorithm, an STD Block is designed to reduce the loss of features during down-sampling by saving spatial information to the channel dimension, and the CARAFE operator, which increases the feature map size without changing the feature mapping mean, is adopted to ensure that features are not distorted by relational scaling. In addition, in order to make full use of the detailed features extracted by the backbone network, the neck network is improved in this study so that the feature extracted after one down-sampling of the backbone network is fused with the top-level semantic information by the neck network to obtain the target detection head with a small receptive field. The experimental results show that the YOLO-FR model proposed in this paper achieved 97.4% on mAP50, which is a 7.4% improvement compared to the original network, and it also outperformed J-MSF and YOLO-SASE.

## 1. Introduction

Infrared dim-small target detection is a key and difficult point in infrared detection technology. It is used in a wide range of scenarios and is a key technology for various industrial applications, such as precision navigation and security monitoring. The geometric and textural structure of infrared dim-small targets is extremely scarce, and noise interference, such as clouds, waves, ground buildings, and human interference, often occur in practical application scenarios, which brings great obstacles to the detection of infrared weak and small targets.

Traditional infrared small target detection algorithms can be divided into two types, namely geometry-based detection algorithms and statistical-based detection algorithms. The former distinguishes the target from the background by the inherent patterns and changing characteristics of the image, while the latter extracts the target feature points from the image based on the assumption that the target fits the designed model. Geometry-based detection algorithms include the morphological filter-based detection method [1,2], the wavelet transform-based detection method [3], the matched filter-based detection method [4], the pipeline filter-based detection method [5], the optical flow method [6,7], and the human visual system-based detection method [8,9]. Statistical-based detection algorithms include scale-invariant feature transform (SIFT) [10], histogram of oriented gradient (HOG) [11], deformable part model (DPM) [12], and other frameworks based on sliding windows and manual feature extraction. Traditional target detection algorithms are still applied widely in the field of infrared small target detection today. Hu [13] divided marine debris detection into two steps—anomalous spatial detection, and anomalous pixel visible and near-infrared spectral band analysis—and achieved good detection results, but the spectral library of marine debris is not perfect, which makes it impossible to distinguish the species to which some marine debris belongs. Based on the infrared speckle tensor (IPT) model, Cao et al. [14] designed a new vector form of the tensor to better exploit the hidden information between different modes of the tensor, which can better suppress the background and detect small infrared targets in complex scenes but can be time-consuming in some complex scenes. Both algorithms mentioned above perform target detection based on manually designed or computed features, so they are less robust and less generalizable.

To improve the robustness and generalizability, scholars use deep neural networks, which allow the model to learn the features needed for the task during training, instead of relying on artificially prescribed features. Since AlexNet [15], designed by Hinton, won the ImageNet competition in 2012 and significantly refreshed the algorithm performance, related algorithms based on deep neural networks have been developed over the years and have become the mainstream approach in computer vision. Scholars have performed many works related to target detection using deep neural networks [16,17,18]. Faster R-CNN [19], SSD [20], and the YOLO framework [21,22,23,24,25,26] are the most representative deep learning target detection frameworks nowadays, demonstrating good performance over traditional algorithms on various datasets. R-CNN and SDD belong to a two-stage detection algorithm, which focuses on detection accuracy but a low detection rate, and YOLO belongs to a one-stage algorithm, which can achieve a high detection rate but will sacrifice certain detection accuracy.

In order to improve the detection accuracy of deep learning methods, a number of studies on improved methods have emerged. Some scholars have enhanced the model’s performance by improving the model’s ability to extract features and output deeper features. Zhou et al. [27] used a migration learning strategy to fine-tune a pre-trained AlexNet model with small sample data in order to retain the powerful feature extraction capabilities obtained by training the model with a large-scale dataset and to enable the model to identify earth and rock embankment leakage features in infrared images. Fu C Y et al. [28] proposed the DSSD framework, which replaced the VCG-16 backbone network of SSD with ResNet-101, and the deconvolutional module and prediction module were added to improve the model’s ability to recognize and classify small targets. Liu et al. [29] proposed UAV-YOLO, which is based on YOLOv3 and was first optimized by connecting two ResNet units of the same width and height to Resblock in the DarkNet network. They also enhanced the feature extraction capability and enriched the spatial information by adding convolution operations in the early layers, in which the mAP outperformed the original algorithm in the small target detection task from the UAV viewpoint.

Feature integration is also an effective way to improve model performance. Lin et al. [30] proposed feature pyramid networks (FPNs) based on Faster R-CNN networks, which fuse high-dimensional semantic information with low-dimensional detail information to solve the problem of serious feature loss of small targets after operations such as multilayer pooling. On this basis, Gong Y et al. [31] pointed out that the top-down connection of adjacent layers in FPNs resulted in two-sided effects on the detection of small targets instead of purely positive ones, and proposed the method of fusing factor weights to control the ratio of information transfer from deep to shallow layers to ensure the fusion of more positive information. Hong et al. [32] designed SPPNet for feature integration in small target detection by discarding the bidirectional fusion approach of FPNs. SPPNet solves the problem of inconsistent gradient calculation between different layers in FPNs and has better performance in the small target detection task. Different from the layer-by-layer integration mechanism of FPNs and their improved methods, Zheng et al. [33] created the CSF module for the small target detection of coconut crowns. The CSF module integrates features at different levels together by first concatenating and then convolving them to connect the shallow and deep-level semantic features.

However, the sampling method in the model also affects the performance of small target detection, which is of research significance. The network usually needs to reduce the feature map size by down-sampling to reduce the model parameters and needs to align feature maps of different sizes by up-sampling for feature fusion. The mainstream models today often use stride = 2 convolutions for down-sampling and the nearest neighbor method, bilinear interpolation, or deconvolution [34] for up-sampling. The shortcomings of these sampling methods have a large impact on the detection of small targets. For example, stride = 2 convolutions will discard half of the features and cause asymmetric sampling, and both the nearest neighbor method and the bilinear interpolation method will cause distortion of the pixel geometric features.

In order to reduce the loss of features in the sampling stage, some improved sampling methods have been proposed. Zhang [35] pointed out that the traditional down-sampling method ignored the sampling theorem, and based on the idea of low-pass filtering before down-sampling, he designed MaxBlurPool, ConvBlurPool, and BlurPool and conducted experiments on various networks to prove their effectiveness. Mazzini [36] designed the GUM to improve up-sampling kernels by learning conversions based on high-resolution details and achieved better up-sampling results.

Pixel shuffle [37] and CARAFE [38] are also important up-sampling methods.

Pixel shuffle is a classical up-sampling algorithm that implements sub-pixel convolution with stride = 1/r (r is the up-sampling factor) to extract the feature map and then expands the obtained feature map by a dimensional space to obtain the up-sampling result. This algorithm scales the image size without changing the current amount of feature information. The sampling idea is beneficial for the detection of small targets, and the down-sampling method in this paper is designed following this idea.

CARAFE is a region content-based up-sampling method that first obtains the up-sampling kernel set by the up-sampling kernel prediction module, and then uses the up-sampling kernel set to up-sample the corresponding positions of the original map. This method outperforms the traditional interpolation method in terms of effectiveness, and the number of parameters is much smaller than that of deconvolution. Assuming that the up-sampling ratio is σ and the size of the input feature map is H×W×C, the CARAFE operator first predicts the set of up-sampling kernels of size $\sigma H\times \sigma W\times K_{up}^{2}$ by the up-sampling kernel prediction module, where $K_{up}^{2}$ is the size of a single up-sampling kernel. Then, the feature reassembly module uses the predicted up-sampling kernel to complete the up-sampling and obtains the output feature size as σH×σW×C. Due to the normalization operation of the up-sampling kernel prediction module, the mean value of features in the region after up-sampling is guaranteed to be constant, which can reduce the feature distortion in the up-sampling process; this is important for small target detection. Zhang et al. [39] used CARAFE in a SAR ship instance segmentation, and the small ship instance segmentation performance was significantly improved.

This paper proposes a complex background infrared dim-small target detection method based on feature reassembly sampling methods. The main contributions are as follows:

1. For the down-sampling process, an STD block was designed to down-sample the image. This method can transfer more space domain information to the depth dimension, which is beneficial for the extraction of small target features and will not lead to an increase in parameters. The STD block is used to complete all the down-sampling operations of the backbone network;
2. For the up-sampling process, the CARAFE operator is used to complete the up-sampling operation in the feature fusion network. The evaluation metrics and visualization results show a significant improvement in the detection accuracy of the model for small targets after using the CARAFE operator;
3. In order to use more shallow detailed features to find small targets, the feature fusion network was expanded to output the features extracted from the backbone network after a down-sampling for fusion, a small target detection head with a smaller receptive field was added, and experiments were designed to find the best target detection head combination.

The rest of this paper consists of the following: Section 2 introduces the YOLO-FR network structure. Section 3 describes the dataset used in this study, the experimental environment, and the evaluation metrics and presents the experimental results and analysis. Section 4 discusses the differences between YOLO-FR and other existing algorithms and summarizes the experimental shortcomings and outlook. Finally, Section 5 concludes the whole paper.

## 2. YOLO-FR

In order to improve the detection capability of the model for infrared dim-small targets, the sampling method for the network was improved in this study, using shallow detail features to obtain a target detection head with a smaller receptive field. This section first introduces the network structure of YOLO-FR, then the improvement of the sampling method, and finally, the use of shallow detail features.

### 2.1. YOLO-FR Network Structure

YOLO-FR takes the YOLOv5s network as the basic model, which can be summarized in three modules: the backbone network, the neck network, and the target detection head. The backbone network is used to extract image features, the neck network fuses the feature output from the backbone network, and the target detection head compresses the channel size to 3*(5+class_num) by using a convolutional layer for the feature map after the feature fusion network. This size means that one feature point is used to predict three bounding boxes, five parameters are used to predict the position size of the bounding box (x,y,w,h) and the confidence level of the target (*C*), and one parameter is used to predict the probability that the target belongs to each class, whose size is consistent with the number of classes in the dataset. Then, the compressed features are used to predict the target boxes, and CIOU-NMS [40] is used to calculate the loss.

**Figure 1:**
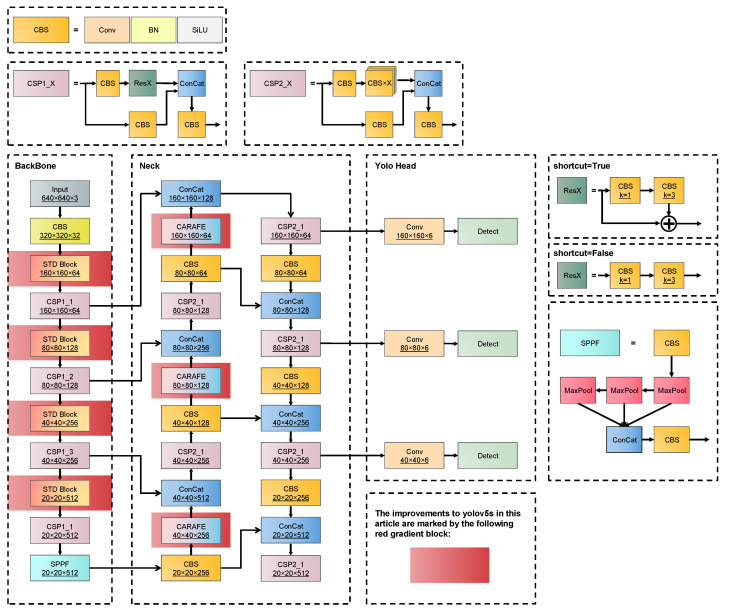
YOLO-FR model network framework.

Based on the idea of feature reassembly, YOLO-FR improves the sampling process of the backbone and neck networks by designing the STD Block in the backbone network to complete the down-sampling of the feature maps and using the CARAFE operator in the neck network for up-sampling. In addition, to obtain more small target detail features, the neck network is extended, and the target detection head with a smaller receptive field is obtained by fusing the features extracted after a single down-sampling of the backbone network. Experiments were designed to find the best combination of target detection heads. The YOLO-FR network framework is shown in Figure 1, with improvements marked by red gradient blocks.

#### 2.1.1. STD Block-Based Feature Reassembly Down-Sampling

YOLOv5s uses stride = 2 convolutions for down-sampling, which reduces half of the convolution operation compared to maxpooling and is faster, but at the same time, half of the features are discarded, resulting in permanent loss of information at certain locations, which is very unfavorable for the detection of small-sized targets. Moreover, this operation samples every two rows/columns, so the number of samples of odd row/column features is not equal to the even row/column features, which will cause distortion of the features.

**Figure 2:**
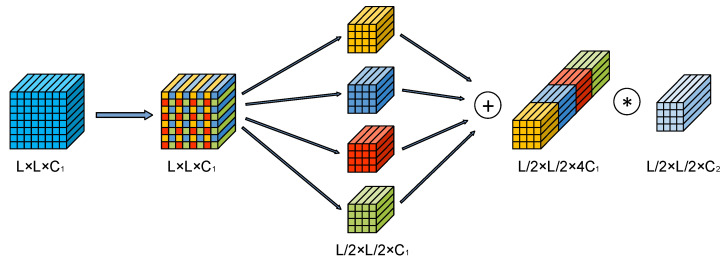
The operation process of the STD Block when scale=2.

In order to retain more small target features in the down-sampling process, an STD Block was designed. The STD Block consists of an STD layer and a convolutional layer with stride = 1. The STD layer down-samples the image by using the space-to-depth algorithm and controls the down-sampling multiplier by the parameter scale, which was inspired by the pixel shuffle up-sampling method [37] and can be considered as the pixel shuffle reverse process. The convolutional layer with stride = 1 performs channel compression on the down-sampling results obtained from the STD layer and shrinks to the target number of channels. Figure 2 shows the operation process of the STD Block with scale=2.

Suppose the input image X size is $L\times L\times C_{1}$. The STD layer first slices the image, and four sets of sequences can be obtained, all of which are in size $L/2\times L/2\times C_{1}$. The sliced sequences can be expressed as Equation (1), and the formula can be summarized as $f_{i,j}=X\left[i:L:2,\;j:L:2\right]$, which means that the pixel point with coordinates (i,j) in the input image is taken as the starting point, and one pixel point is taken every two columns until the image boundary. The operation is repeated every two lines to form the final sequence $f_{i,j}$. The obtained slice sequence is concatenated along the channel dimension to obtain the intermediate feature with the dimensions of $L/2\times L/2\times 4C_{1}$.

(1) $$\begin{array}{c} \begin{array}{c} f_{0,0}=X\left[0:L:2,\;0:L:2\right]\\ f_{0,1}=X\left[0:L:2,\;1:L:2\right] \end{array}\\ \begin{array}{c} f_{1,0}=X\left[1:L:2,\;0:L:2\right]\\ f_{1,1}=X\left[1:L:2,\;1:L:2\right] \end{array} \end{array}$$

**Figure 3:**
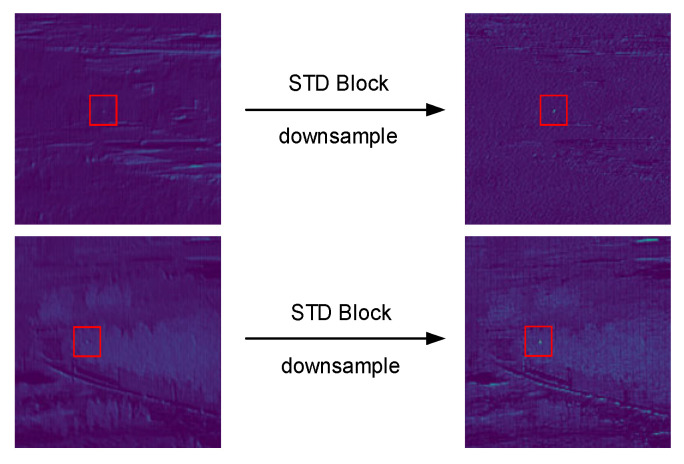
Down-sampling results of STD Block.

The features are extracted from the intermediate result obtained from the STD layer by using a convolutional layer with stride = 1 and channel dimension = $C_{2}$, whose channels are compressed to the output size. Compared to the convolution with stride = 2, the convolution layer with stride = 1 can better retain the discriminative feature information. The results of down-sampling the feature maps using the STD Block are shown in Figure 3, the red box marks the area where the target is located.

#### 2.1.2. Feature Reassembly Up-Sampling Based on CARAFE Operator

There are many up-sampling methods available in YOLOv5s, among which the nearest neighbor method and the bilinear interpolation method interpolate through the spatial relationship of existing pixels, which are simple to implement, but the former will change the geometric continuity of the image element values, and the latter will cause the edges to be smoothed, neither of which can effectively maintain the features. Although deconvolution can reduce feature distortion through parameter learning, it inevitably introduces a large number of parameters. In order to maintain features during up-sampling without causing a parameter spike, this study used the CARAFE operator for up-sampling.

**Figure 4:**
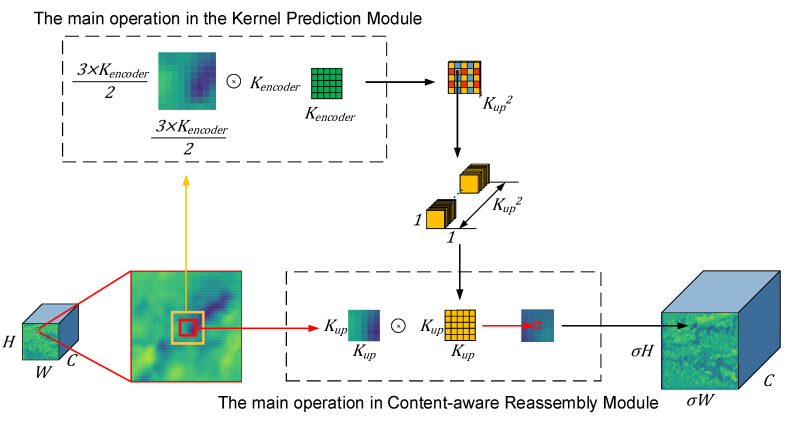
Up-sampling results of feature maps at different $k_{up}$ values.

**Figure 5:**
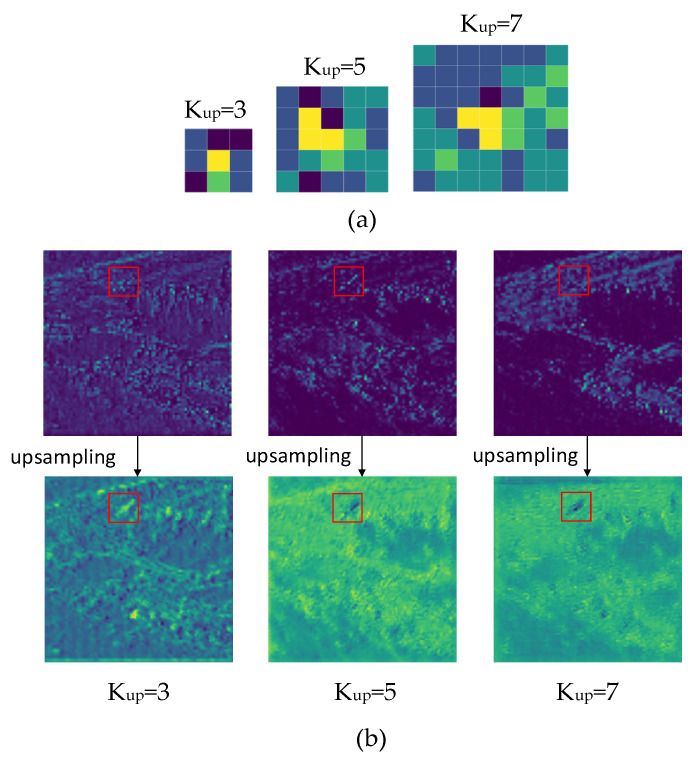
Set $k_{encoder}=k_{up}=k$. When k was different, the results sampled on CARAFE were different. (**a**) The up-sampling kernel obtained from the same center under different k values. (**b**) The results of sampling on the feature map under different k values.

- As shown in Figure 4, the use of the CARAFE operator requires the determination of two parameters, $k_{up}$ and $k_{encoder}$. $k_{up}$ affects the size of the up-sampled kernels in the content-aware reassembly module after expansion. The larger the $k_{up}$, the larger the context area used for up-sampling. $k_{encoder}$ affects the size of the context region used to generate the up-sampled kernels. $k_{up}$ and $k_{encoder}$ should be increased together to ensure that more region information is used. The up-sampling results obtained using different sizes are shown in Figure 5.
- In Figure 5, (a) is the up-sampling kernel generated for the region centered at (160, 70) in the 160 × 160 feature map. The red box in (b) marks the area where the target is located, it can be seen that when the $k_{up}$ is larger, the background features are smoother and the target features are more easily highlighted. However, increasing $k_{up}$ and $k_{encoder}$ will result in an increase in the number of parameters and GFLOPS. When $k_{up}$ was increased from 3 to 5, the number of parameters increased by 139,392 and the GFLOPs increased by 4.5; when $k_{up}$ was increased from 5 to 7, the number of parameters increased by 454,848 and the GFLOPs increased by 14.5. So, in this study, we took $k_{encoder}=k_{up}=3$.

#### 2.1.3. The Use of Shallow Detail Features

**Figure 6:**
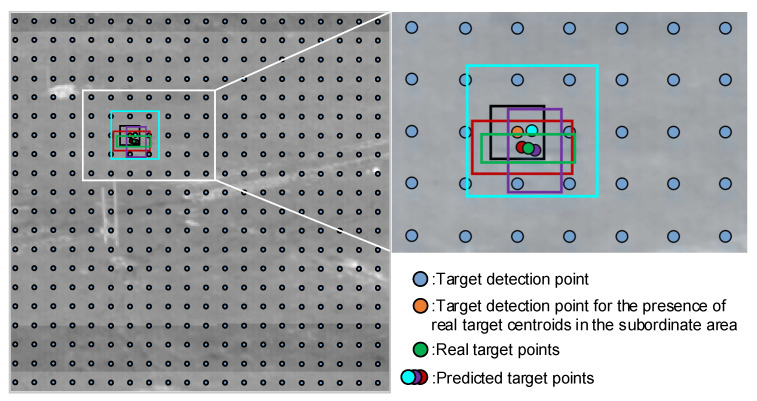
Target box prediction by YOLO head.

- Table 1 shows the names, dimensions, number of down-sampling, and receptive field of the feature maps obtained from the backbone network.
- YOLOv5s is a general target detection model that needs to consider the target detection performance of large, medium, and small sizes simultaneously, so the model has three target detection heads with different receptive fields to perform multi-scale detection of images. However, the target detection head used for small target detection in the original network uses Feature2 to fuse Feature3 and Feature4 with a theoretical receptive field of 5 × 5, which is prone to miss detection when the target size is smaller than 5 × 5. Therefore, Feature1 extracted from the backbone network is put out, and the features are fused with the high-level semantics through the neck network, and then transformed into the small target detection head through a convolution layer.
- According to their size, the four target detection heads are named YOLO head 1, YOLO head 2, YOLO head 3, and YOLO head 4 from large to small, respectively. Taking YOLO head 1 as an example, the predicted target boxes are shown in Figure 6. The YOLO head divides the image into 20 × 20 cells by 20 × 20 feature points. When the target center point within the label falls within a certain cell, such as the area where the yellow circle is located in Figure 6, three target boxes (red, cyan, and purple dots in Figure 6) are predicted, including the center position of the target box, the width, and height. During training, the localization loss is calculated using CIoU_Loss, and the parameters are updated using the stochastic gradient descent method. When the YOLO head size is larger, the receptive field is smaller, but the image is split into more copies, which is suitable for the detection of small targets. Multi-scale detection requires the use of a combination of YOLO heads.
- In this study, experiments were designed to find the best combination of YOLO heads. The experimental design concept is to determine whether the small YOLO head contributes to the detection of infrared dim-small targets and to identify the effect of a YOLO head with a large receptive field on the detection of infrared dim-small targets. The combinations of the target detection heads involved in the experiments are shown in Table 2, and the experimental results and analysis are given in Section 3.

## 3. Experiments and Results

### 3.1. Dataset

The dataset used in this study was the infrared dim-small airplane target dataset publicly available from Liu et al. [41]. There were 22 sets of data in the dataset, totaling 16,177 infrared images, all with an image size of 256 × 256. To avoid the network learning a large number of single features during the training process, which may lead to overfitting, images with a high signal-to-noise ratio and single features in the dataset needed to be eliminated.

**Figure 7:**
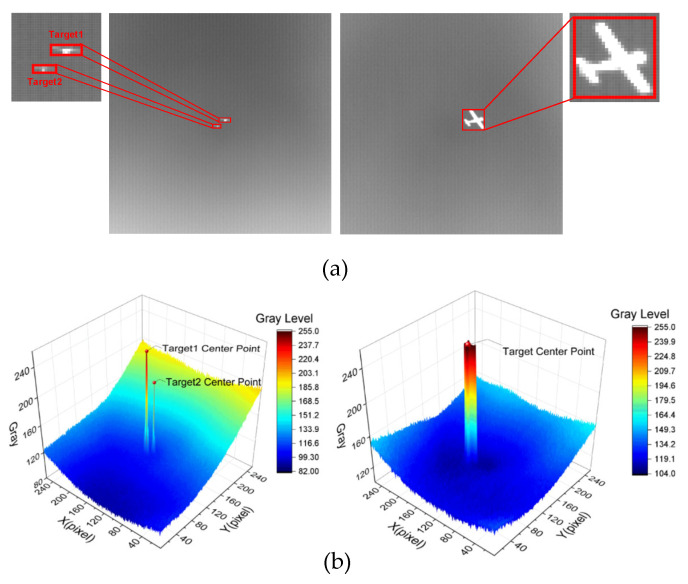
Example of infrared images to be deleted. (**a**) Infrared image and target magnification. (**b**) 3D surface map based on the pixel grayscale of (**a**).

The excluded data are shown in Figure 7, where (a) marks the target with the red box and zooms in; (b) plots the gray value of each point in the original image as a 3D surface map, where X indicates the horizontal coordinate, and Y indicates the vertical coordinate; grayscale indicates the gray value at the current coordinate, and the gray level converts the gray value into a color level. As can be seen in (b), the two images have a large signal-to-noise ratio, the background is very smooth, and the grayscale distribution is clearly separated from that of the target.

**Figure 8:**
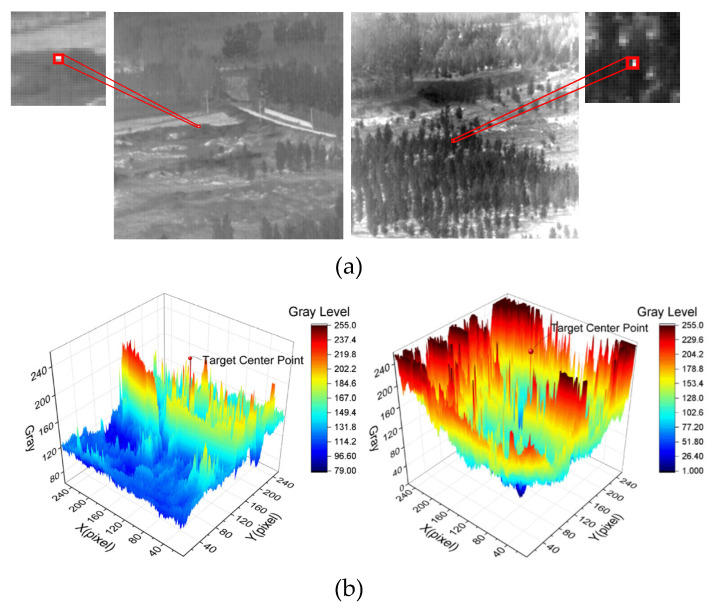
Example of retained infrared images. (**a**) Infrared image and target magnification. (**b**) 3D surface map based on the pixel grayscale of (**a**).

Finally, 2102 infrared images were deleted, and the retained images all conformed to the characteristics of small infrared targets in complex backgrounds, as shown in Figure 8.

**Figure 9:**
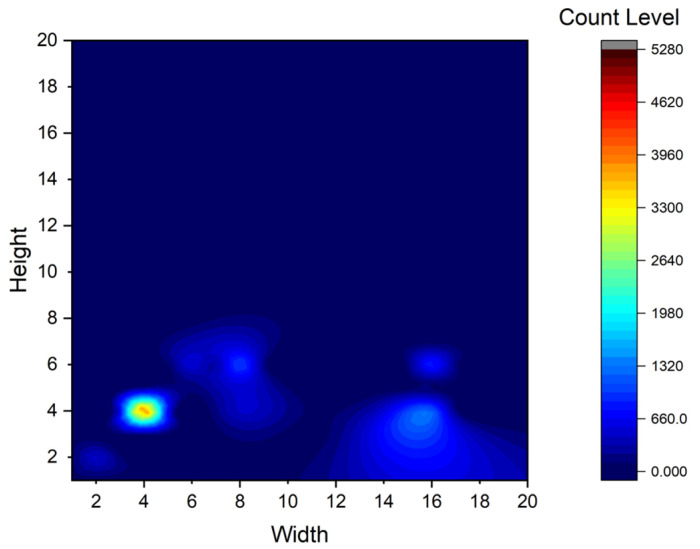
Target box size distribution.

The size distribution of the retained image was visualized and is shown in Figure 9, in which the horizontal coordinate indicates the width of the target box, the vertical coordinate indicates the length of the target box, and the Count Level indicates the color level corresponding to the number of target boxes with corresponding length and width.

### 3.2. Experimental Environment

The software environment and hardware parameters used for the experiments are shown in Table 3. There are major differences between Python 3.x and Python 2.x. The main differences are the data types and encoding methods. Python 2.x defaults to ASCII encoding, while Python 3.x uses UTF-8, which facilitates more language encoding.

The parameters of the training YOLO-FR are shown in Table 4.

The dataset was split on the fly according to a ratio of 4:1, where 4/5 of the data were used as the training set and 1/5 of the data were used as the test set. Then, 1/5 of the data from the training set were taken as the performance validation set during training.

### 3.3. Evaluation Indicators

Precision, recall, and mean average precision (mAP) are used as the evaluation metrics for network performance. Both precision and recall are calculated based on the confusion matrix, which is shown in Table 5. Since no negative samples exist in the dataset, FN = 0 in the confusion matrix. Precision indicates the proportion of correct target boxes in the predicted target box, as shown in Equation (2). Recall indicates the ratio of the correct target boxes detected by the network to the total number of true correct target boxes, as shown in Equation (3). Since the detection is the single type, the mAP is equal to the average precision of the unique class, which is equal to the area enclosed by the precision–recall curve (P-R) and the coordinate axis, as Equation (4) shows.

(2) $$Precision=\frac{TP}{TP+FN}$$

(3) $$Recall=\frac{TP}{TP+FP}$$

(4) $$mAP=AP=\int_{0}^{1}P\left(R\right)dR$$

In addition to assessing the model performance by using the confusion matrix, the performance differences among models were visualized by comparing the detection results of the network on classical samples, with the following meanings of the target boxes in different colors in the figure:

- The red target box indicates the location of the target detected by the model. The wrong target is indicated by “error”, and the target type and confidence level are displayed above the red target box. The only target type for this experiment is “airplane”, and the confidence level is between 0 and 1;
- The green target box indicates the location of the real target, and the target type is displayed above the green target box;
- The blue target box indicates the location of the real target that is not detected by the model;
- The image uses a white box to mark the area where the target appears and is locally zoomed in to show the target location more clearly.

### 3.4. Results and Analysis

#### 3.4.1. Comparative Experiment of Sampling Methods

This section compares the results of different sampling methods on network performance, including the comparison between STD Block down-sampling and stride = 2 convolutional down-sampling, and the comparison between the CARAFE up-sampling method and the neighborhood interpolation up-sampling method. In the comparison experiments, the down-sampling in the backbone network was carried out by an STD Block and a convolution of stride = 2, and the up-sampling in the neck network was carried out by CARAFE and neighborhood interpolation, while the rest of the modules remained unchanged.

**Figure 10:**
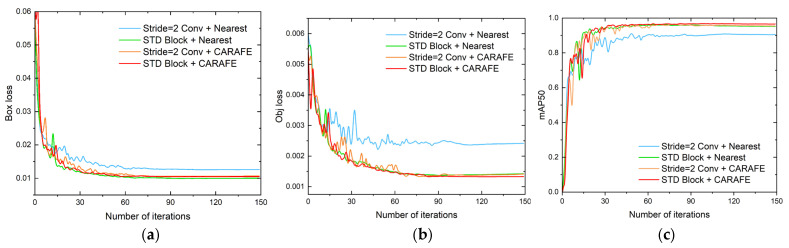
Variations in mAP and loss with the number of iterations for different sampling methods. (**a**) box loss. (**b**) object loss. (**c**) mAP50.

The changes in the loss and mAP50 during the training process are shown in Figure 10. It can be seen that the loss of the model tended to be stable when training 150 epochs, and the loss of the model based on the feature reassembly sampling method was significantly lower than that of the model based on the original method. The results of the sampling comparison are shown in Table 6.

As can be seen from the results in Table 6, when down-sampling with an STD Block in the backbone network alone or up-sampling with CARAFE in the neck network alone, the improvement in precision was ≥2.4%, the improvement in recall was ≥11.2%, and there was a 6.4% improvement in the mAP50 obtained in both ways. When sampling with the STD Block and CARAFE at the same time, the precision was improved by 2.4%, the recall was improved by 11.7%, the mAP50 was improved by 6.9%, and the mAP50-90 was improved by 6.9%, compared to the original method. All performance metrics, except for precision, were maximized when using both STD Block and CARAFE, and the recall improvement was the largest, which proves that the sampling method based on feature reassembly had a significant positive effect on extracting the features of small targets.

**Figure 11:**
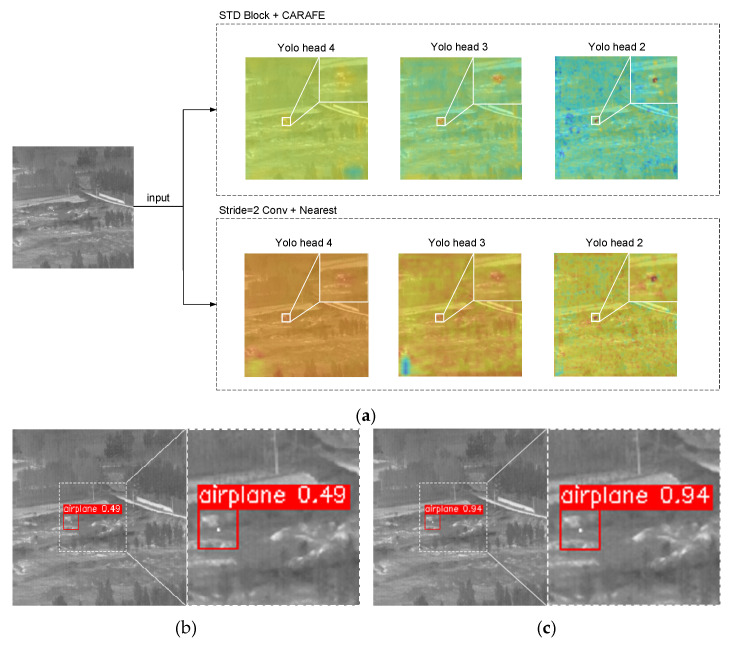
Heat map of YOLO head and model detection results obtained using the feature reassembly sampling method and the traditional sampling method. (**a**) Heat map of YOLO head with different sampling methods. (**b**) Detection results using STD Block + CARAFE. (**c**) Detection results using stride = 2 convolutions + nearest.

In order to analyze the effect of the sampling method on small target detection more intuitively, this study referred to CAM [42] and compared the effect of stride = 2 convolutions + nearest with STD Block + CARAFE on target detection, and a heat map for each detection head was visualized with the object confidence as the weight. The comparison is shown in Figure 11.

With the use of the feature reassembly sampling method, the detection head paid more attention to target-related information and significantly less attention to noisy information. Figure 11b,c shows the prediction results of the network using different sampling methods, in which the STD Block + CARAFE sampling method obtained prediction results with a confidence of 0.94, while the confidence of the original method was only 0.49.

#### 3.4.2. Comparative Experiment of Different YOLO Head Combinations

This section compares the effects of different combinations of target detection heads on the detection performance. In the comparison experiments, the sampling methods used were STD Block + CARAFE, and the target detection heads included YOLO heads 1~4. The relevant information is the same as shown in Table 2.

**Figure 12:**
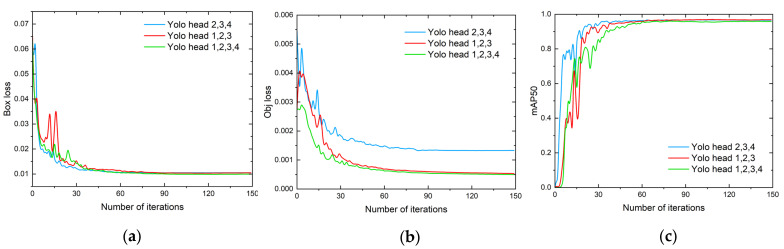
Variations in mAP and loss with the number of iterations for different target detection head combinations. (**a**) Box loss. (**b**) Object loss. (**c**) mAP50.

The changes in the loss and mAP50 during the training process are shown in Figure 12. It can be seen that the loss of the model tended to be stable when training 150 epochs. After using YOLO head 1, the Obj loss decreased significantly compared to the original method. The comparison results are shown in Table 7.

From the comparison results in the table, it is clear that each evaluation index was the highest when using YOLO head 1, YOLO head 2, and YOLO head 3 as a combination. In order to compare the differences more visually, the results of different combinations of detection heads are visualized in a heat map.

**Figure 13:**
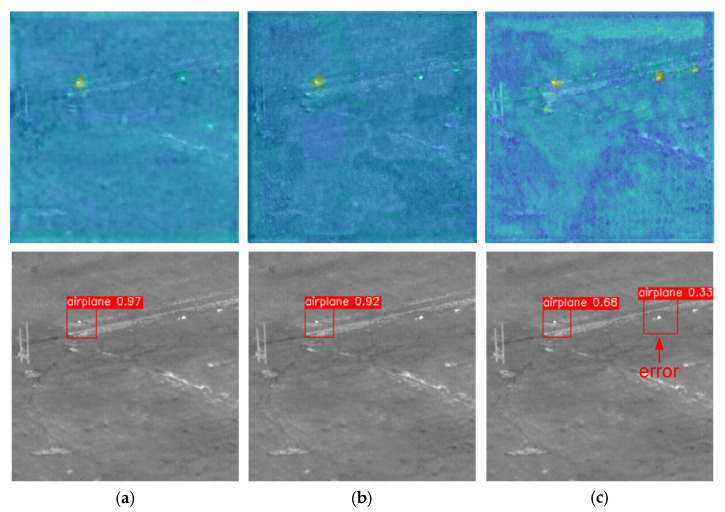
Heat map and detection results of different YOLO head combinations for relatively large-sized targets. (**a**) Combination of YOLO head 2 + YOLO head 3 + YOLO head 4. (**b**) Combination of YOLO head 1 + YOLO head 2 + YOLO head 3. (**c**) Combination of YOLO head 1 + YOLO head 2 + YOLO head 3 + YOLO head 4.

From the heat map in Figure 13c, we can see that the combination of YOLO heads 1, 2, 3, and 4 could focus on more feature information compared to the other two combinations, but due to the complex background with more interference points, many noise highlights formed near-target features with the background, which resulted in a false alarm. The target box marked with a confidence level of 0.33 on the right side of (c) is the false alarm.

**Figure 14:**
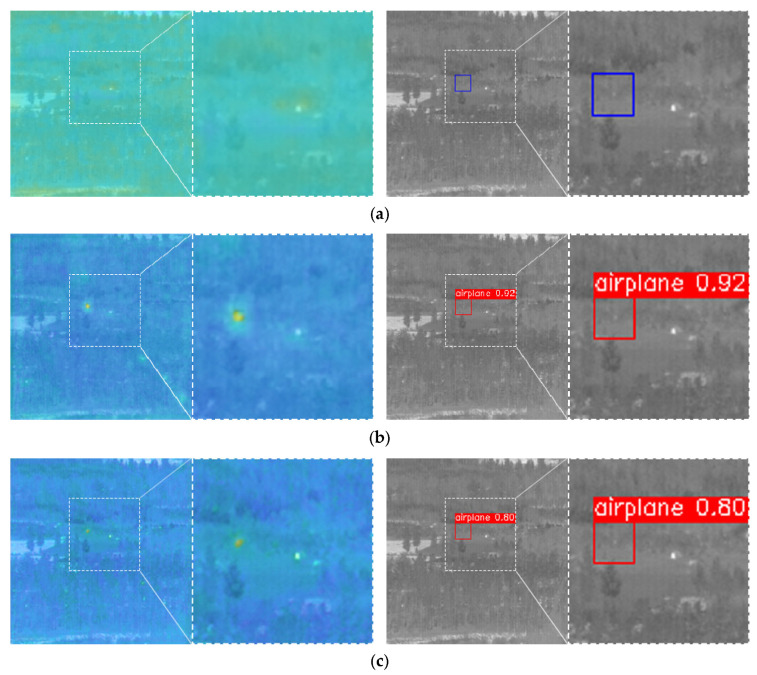
Heat map and detection results of different YOLO head combinations for relatively small-sized targets. (**a**) Combination of YOLO head 2 + YOLO head 3 + YOLO head 4. (**b**) Combination of YOLO head 1 + YOLO head 2 + YOLO head 3. (**c**) Combination of YOLO head 1 + YOLO head 2 + YOLO head 3 + YOLO head 4.

Figure 14a shows the output heat map and detection results under the combination of YOLO heads 2, 3, and 4, respectively. The results of the heat map show that the network did not pay attention to the information related to small targets and no small targets were detected. This combination was weaker than the other two combinations in terms of the detection ability of tiny targets.

From all of the experimental results above, it can be concluded that the combination of YOLO heads 1, 2, and 3 was the most suitable for the infrared dim-small target detection task.

#### 3.4.3. Comparison Test of Different Networks

In order to further verify the superiority of the improved network for infrared small target detection, the classical lightweight models, such as YOLOv3-tiny, YOLOv4-tiny, YOLOv5s, and J-MSF, were compared [43].

**Figure 15:**
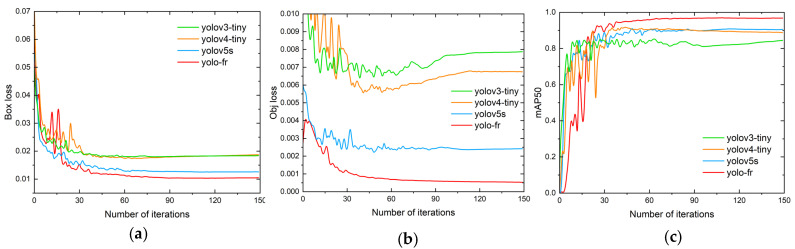
Variations in mAP and loss with the number of iterations for different modules. (**a**) Box loss. (**b**) Object loss. (**c**) mAP50.

The changes in the loss and mAP50 during the training process are shown in Figure 15. It can be seen that the loss of the model tended to be stable when training 150 epochs. It can be seen that the loss and mAP of YOLO-FR were both significantly improved compared to YOLOv3-tiny, YOLOv4-tiny, and YOLOv5s.

The size, number of parameters, and GFLOPS of different models are shown in Table 8, and the performance metrics of different model prediction results are shown in Table 9.

As can be seen in Table 8, YOLO-FR had a smaller number of parameters than YOLOv3-tiny, and the model size only increased by 3.2 MB compared to YOLOv5s. However, the image-splitting operation of the STD Blocks led to a substantial increase in GFLOPS, which was 2.09 times larger than the original model. To avoid the further growth of GFLOPS, YOLO-FR only used STD Blocks for down-sampling in the backbone network, while the stride = 2 convolutions was still used for down-sampling in the neck network.

**Figure 16:**
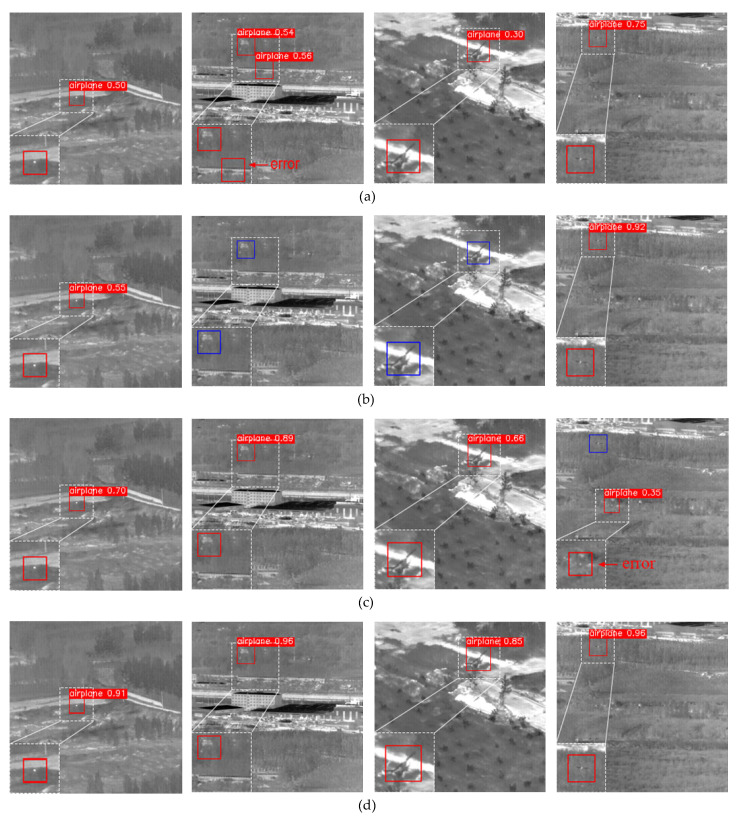
Detection results of different models. (**a**) YOLOv3-tiny. (**b**) YOLOv4-tiny. (**c**) YOLOv5s. (**d**) YOLO-FR.

As is shown in Table 9, YOLO-FR had the highest mAP50 among these networks and far exceeded the classical lightweight model in terms of precision, recall, and mAP. Although it was slightly lower than J-MSF in terms of recall, the improvement effect of YOLO-FR in precision was significantly better than that of J-MSF. The final size of the model was 16.9 MB, indicating that the model is deployable on embedded platforms while being more suitable for infrared dim-small targets. Four different scenarios were selected to show the detection results of each network in Figure 16. The red box in the figure is the target detected by the model, the wrong target is indicated by “error”, and the black box indicates the real target which is not detected. It can be seen that YOLO-FR had better performance in all four scenarios.

## 4. Discussion

**Figure 17:**
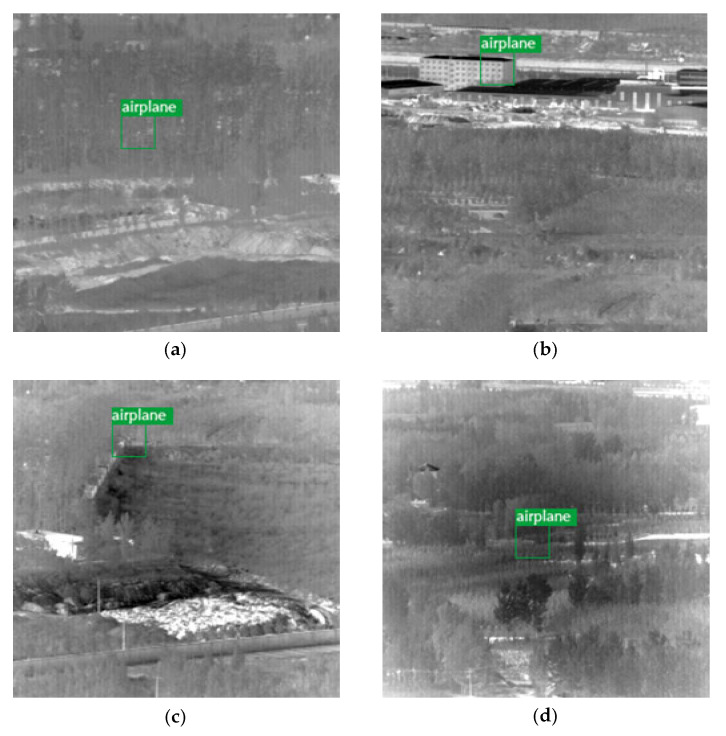
Example of four sets of low signal-to-noise ratio images in a dataset. (**a**) Data13 sample example. (**b**) Data14 sample example. (**c**) Data17 sample example. (**d**) Data21 sample example.

Zhou et al. [44] proposed YOLO-SASE, using YOLOv3 as the base framework, reconstructed the input images using super-resolution, and added the SASE module to the network to improve the stability of the model. In their paper, they pointed out the existence of difficult-to-detect low signal-to-noise ratio data in this dataset, including Data13, Data14, Data17, and Data21, and the sample examples are shown in Figure 17, where the green box marks the real position of the target. The precision of YOLO-SASE for recognizing this part of the data was 68.95%, and the recall rate was 61.73%.

**Figure 18:**
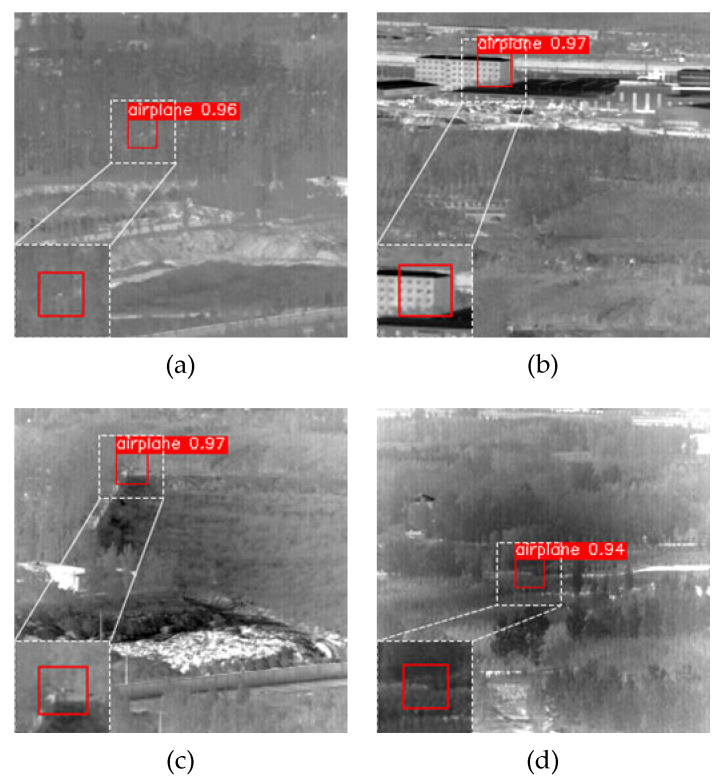
Detection results of the YOLO-FR model for four groups of hard-to-detect sample examples. (**a**) Data13 sample example. (**b**) Data14 sample example. (**c**) Data17 sample example. (**d**) Data21 sample example.

The detection results of YOLO-FR on the same dataset of 630 images were compared with those of YOLO-SASE, as shown in Table 10. From the table, it can be seen that the model was significantly better than YOLO-SASE in terms of precision and recall, which shows that the model had a better detection effect on the low signal-to-noise ratio samples that were difficult to detect with YOLO-SASE. The detection results of this paper’s model for the examples are shown in Figure 18.

**Figure 19:**
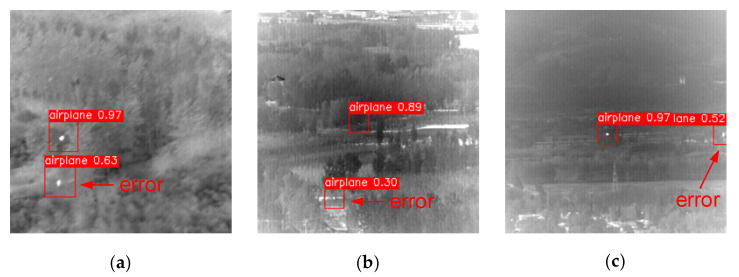
Detection of false alarm scenarios. (**a**–**c**) Detection of other bright spots in the figure as targets, resulting in false alarms.

Although YOLO-FR had better results compared to J-MSF and YOLO-SE in the same scenario, it also had its own limitations. First, the stronger small target detection capability of YOLO-FR greatly improved recall, but it also introduced a false alarm in some scenes, as shown in Figure 19. However, these identified vignettes did not have multi-frame continuity and could therefore be excluded by multi-frame filtering.

In addition, due to the limited IR dataset, the size of the training dataset in this study was not sufficient to support YOLO-FR to provide transfer learning to other models. Two approaches can effectively improve the transferability of the model. The first one, based on the partial domain adaptation approach proposed by Zheng [45] et al., optimizes YOLO-FR by transferring other models that contain small target detection. The other one trains YOLO-FR by using a larger number and a larger variety of small IR target images.

## 5. Conclusions

This paper proposes YOLO-FR, an improved model of YOLOv5s based on the feature reassembly sampling method, which improves the infrared dim-small target detection capability of the model by reducing the loss of small target features in the sampling phase. In this algorithm, an STD Block is designed to complete the down-sampling in the backbone network, and the CARAFE operator is used to up-sample the feature maps in the neck network. The STD Block can transform more spatial information into the channel dimension through the space-to-depth algorithm, while the CARAFE operator can reduce the distortion caused by the change in the mapping mean of the features, both of which are beneficial for the model to extract small faint features of the target. In addition, the details extracted from the backbone network were used and the neck network was extended to obtain a small target detection head with a smaller receptive field. Experiments were designed to find the optimal target detection head combination. With the infrared dim-small airplane target dataset used as the experimental dataset, the results show that YOLO-FR outperformed the compared models in terms of precision, recall, and mAP.

## Figures and Tables

**Table 1:** Name, size, number of down-sampling, and sensory field correspondence of feature maps obtained from the backbone network.

Name	Size	Number of Down-Sampling	Sensory Field
Feature4	20 × 20	4	9 × 9
Feature3	40 × 40	3	7 × 7
Feature2	80 × 80	2	5 × 5
Feature1	160 × 160	1	3 × 3

**Table 2:** Target detection head combinations involved in the experiment.

Target Detection Head Combinations
YOLO head 2 + YOLO head 3 + YOLO head 4
YOLO head 1 + YOLO head 2 + YOLO head 3 + YOLO head 4
YOLO head 1 + YOLO head 2 + YOLO head 3

**Table 3:** Software environment and hardware parameters.

Platform	Configuration
Integrated development environment	PyCharm
Scripting language	Python 3.8
Operating system	Ubuntu18.04
CPU	Intel(R) Xeon(R) Platinum 8255C CPU
GPU	RTX 2080 Ti
GPU accelerator	CUDA10.1
Memory	43 GB

**Table 4:** YOLO-FR training parameters.

Parameter	Configuration
Neural network optimizer	SGD
Learning rate	0.01
Momentum	0.937
Training epochs	150
Batch size	16

**Table 5:** Confusion matrix.

	True	False
Positive	TP	FP
Negative	TN	FN

**Table 6:** Comparison of experimental results of sampling.

Method	Precision (%)	Recall (%)	mAP50 (%)	mAP50-90 (%)
Stride = 2 Conv + Nearest	93.7%	81.8%	90.0%	72.9%
STD Block + Nearest	96.1%	93.0%	96.4%	79.3%
Stride = 2 Conv + CARAFE	**96.6%**	93.1%	96.4%	78.2%
STD Block + CARAFE	96.1%	**93.5%**	**96.9%**	**79.8%**

**Table 7:** Comparison of experimental results of different head combinations.

YOLO Head Combination	Precision (%)	Recall (%)	mAP50 (%)	Map50-90 (%)
YOLO head 2,3,4	96.1%	93.5%	96.9%	79.8%
YOLO head 1,2,3	**97.0%**	**95.4%**	**97.4%**	**82.6%**
YOLO head 1,2,3,4	95.0%	91.7%	95.6%	80.7%

**Table 8:** Name, size, number of down-sampling, and sensory field correspondence of feature maps obtained from the backbone network.

Method	Model Size (Parameters)	Model Size (MB)	GFLOPS (Forward Pass)
YOLOv3-tiny	8,666,692	16.6	12.9
YOLOv4-tiny	3,058,756	5.96	6.3
YOLOv5s	7,012,822	13.7	15.8
J-MSF	-	-	-
YOLO-FR	8,335,730	16.9	33.1

**Table 9:** Comparison of experimental results of different model prediction methods.

Method	Precision (%)	Recall (%)	mAP50 (%)	mAP50-90 (%)	Inference Speed (ms)
YOLOv3-tiny	89.5	82.2	85.2	60.6	3.0
YOLOv4-tiny	90.1	86.2	90.4	70.2	**2.6**
YOLOv5s	93.7	81.8	90.0	72.9	5.1
J-MSF [43]	88.0	**96.3**	96.2	-	14.8
YOLO-FR	**97.0**	95.4	**97.4**	**82.6**	8.7

**Table 10:** Detection results of YOLO-SASE and YOLO-FR on samples with low signal-to-noise ratios.

Method	Precision (%)	Recall (%)
YOLO-SASE	69.0	61.7
YOLO-FR	95.4	93.7

## Data Availability

These data were derived from the following resources available in the public domain [41]: A dataset for infrared image dim-small aircraft target detection and tracking underground/air background.
